# Estimating the global impact of rotavirus vaccines on child mortality

**DOI:** 10.1016/j.ijid.2023.10.005

**Published:** 2023-12

**Authors:** Andrew Clark, Sarwat Mahmud, Frederic Debellut, Clint Pecenka, Mark Jit, Jamie Perin, Jacqueline Tate, Heidi M. Soeters, Robert E. Black, Mathuram Santosham, Colin Sanderson

**Affiliations:** 1 Department of Health Services Research and Policy, Faculty of Public Health and Policy, London School of Hygiene and Tropical Medicine, London, UK; 2 PATH, Geneva, Switzerland; 3 PATH, Seattle, WA, USA; 4 Department of Infectious Disease Epidemiology, London School of Hygiene and Tropical Medicine, London, UK; Modelling and Economics Unit, Public Health England, London, UK; 5 Department of International Health, Bloomberg School of Public Health, Johns Hopkins University, Baltimore, USA; 6 Centers for Disease Control and Prevention, Atlanta, USA; 7 World Health Organization, Geneva, Switzerland

**Keywords:** Rotavirus, Rotavirus vaccination, Childhood diarrhea, Diarrhea mortality

## Abstract

**Objectives:**

We estimated the global impact of rotavirus vaccines on deaths among children under five years old by year.

**Methods:**

We used a proportionate outcomes model with a finely disaggregated age structure to estimate rotavirus deaths prevented by vaccination over the period 2006-2019 in 186 countries. We ran deterministic and probabilistic uncertainty analyses and compared our estimates to surveillance-based estimates in 20 countries.

**Results:**

We estimate that rotavirus vaccines prevented 139,000 under-five rotavirus deaths (95% uncertainty interval 98,000-201,000) in the period 2006-2019. In 2019 alone, rotavirus vaccines prevented 15% (95% uncertainty interval 11-21%) of under-five rotavirus deaths (0.5% of child mortality). Assuming global use of rotavirus vaccines and coverage equivalent to other co-administered vaccines could prevent 37% of under-five rotavirus deaths (1.2% of child mortality). Our estimates were sensitive to the choice of rotavirus mortality burden data and several vaccine impact modeling assumptions. The World Health Organization's recommendation to remove age restrictions in 2012 could have prevented up to 17,000 rotavirus deaths in the period 2013-2019. Our modeled estimates of rotavirus vaccine impact were broadly consistent with estimates from post-vaccination surveillance sites.

**Conclusion:**

Rotavirus vaccines have made a valuable contribution to global public health. Enhanced rotavirus mortality prevention strategies are needed in countries with high mortality in under-5-year-old children.

## Highlights

• Rotavirus vaccines prevented around 140,000 child deaths in the period 2006-2019.
• Relaxing age restrictions prevented up to 17,000 deaths in the period 2013-2019.
• Global use of rotavirus vaccines could prevent over one-third of rotavirus deaths.

## Introduction

The current generation of live oral rotavirus vaccines have been licensed since 2006 for the prevention of rotavirus gastroenteritis (RVGE) in young children. By the end of 2019 over 100 countries had introduced one of the four rotavirus vaccines currently licensed for global use, and over one-third of the world's infants had received at least two doses [1].

Rotavirus vaccines are widely considered to have had an important impact on under-five RVGE deaths, particularly in low- and middle-income countries (LMICs). However, most LMICs do not have reliable systems for reporting under-five RVGE deaths over time, making it difficult to observe vaccine impact directly. Modeling is therefore needed to estimate the contribution that rotavirus vaccines have made to global public health and the value of continued investment in their uptake. At least three groups have generated independent estimates of the burden of RVGE deaths aged <5 years by country and year [2], and at least three other groups [3] have used these burden estimates as a basis for modeling the impact of rotavirus vaccines on under-five RVGE deaths. The current estimates differ, which can create confusion. Combining available RVGE mortality datasets and exploring the influence of including or excluding different vaccine impact modeling assumptions (e.g., vaccine timeliness, vaccine age restrictions, waning vaccine protection, and indirect effects) may help to build consensus. There are now over 10 years of accumulated data on the real-world impact of rotavirus vaccines from a range of hospital surveillance studies [4,5], and these data can be used to assess the validity of updated estimates.

We aim to provide updated model-based estimates of the global number of under-five RVGE deaths prevented by rotavirus vaccination between January 01, 2006, and December 31, 2019 (a period unaffected by the COVID-19 pandemic), compare them to surveillance-based estimates and assess their sensitivity to different sources of RVGE mortality data and vaccine impact modeling assumptions.

## Methods

We estimated the number of under-five RVGE deaths with and without rotavirus vaccination over the period 2006-2019 in 186 countries and territories defined by the 2022 revision of the United Nations World Population Prospects (UNWPP) [6]. The supplementary appendix (pages 2-6) includes the list of countries that were included and excluded and their 2022-2023 income classification according to the World Bank [7].

We used version 1.7.01 of the UNIVAC decision-support model (www.paho.org/en/provac-toolkit), an Excel proportionate outcomes static cohort model with a finely disaggregated age structure (weeks of age <5 years). In each country, we compared a counterfactual scenario assuming no rotavirus vaccine use to a scenario based on real-world estimates of rotavirus vaccine coverage. All input parameters and their distributions are shown in Table 1. For a given *week* of age between birth and age 5.0 years, the estimated number of RVGE deaths was calculated as:

$$D_{w}*\left(1-\left(C_{3w}*E_{3w}+\left(C_{2w}-C_{3w}\right)*E_{2w}+\left(C_{1w}-C_{2w}\right)*E_{1w}\right)\right)$$

where:

D_w_ = Number of RVGE deaths in *week* of age (without vaccination)
C_1w_ = Coverage of dose one in *week* of age, adjusted for age restrictions
C_2w_ = Coverage of dose two in *week* of age, adjusted for age restrictions
C_3w_ = Coverage of dose three in *week* of age, adjusted for age restrictions
E_1w_ = one-dose vaccine efficacy in *week* of age, adjusted for vaccine waning
E_2w_ = two-dose vaccine efficacy in *week* of age, adjusted for vaccine waning
E_3w_ = three-dose vaccine efficacy in *week* of age, adjusted for vaccine waning

**Table 1:** Parameters and distributions used for base case scenario and probabilistic simulations.^a^

Parameter	Base case (95% CI)	Probability distribution	Source
**Population projections for the 2006-2019 birth cohorts**			
Population by single age/year between birth and 5.0 years	Country-specific	Beta-PERT (mid = UNWPP medium variant, range = UNWPP 2022 revision, low/high variant)	[8]
**Disease burden estimates**			
Rate of RVGE deaths <5 years per 100,000 per year	Country-specific	Beta-PERT (mid = mean of Global Burden of Disease Study, Maternal and Child Epidemiology Estimation Group and World Health Organization and Centers for Disease Control and Prevention country estimates, range = 95% CI)	[9], [10], [11]
**Age distribution of RVGE deaths**			
Log logistic scale (median age) parameter^b^	Country-specific	Beta-PERT (mid = best fit for country/U5MR stratum, range = 95% CI for country/U5MR stratum)	[12]
**Vaccine coverage**			
Rotavirus last dose and Diphtheria-Tetanus-Pertussis drop-out rates	Country-specific	Beta-PERT (mid = WUENIC 2006-2019, range = WUENIC +/-10%)	[1,13]
**Vaccine timeliness**			
Log logistic scale (median age) parameter^b^	Country-specific	Beta-PERT (mid = best fit for country or schedule stratum, range = country interquartile range or median interquartile range for schedule stratum)	[14,15]
**Vaccine efficacy against RVGE mortality**^b^			
Low mortality	99.6% (99.4-100%)	Beta (alpha = 5377, beta = 22, [A] = 0%, [B] = 100%)	[15,16]
Medium mortality	91.4% (89.8-92.7%)	Beta (alpha = 1394, beta = 132, [A] = 0%, [B] = 100%)	[15,16]
High mortality	78.9% (75.5-82.3%)	Beta (alpha = 434, beta = 116, [A] = 0%, [B] = 100%)	[15,16]
**Mean duration of vaccine efficacy in months**^b^			
Low mortality	176.8 (114.7-268.0)	Gamma (alpha = 21.82, beta = 8.41)	[15,16]
Medium mortality	121.9 (81.3-182.4)	Gamma (alpha = 24.01, beta = 5.28)	[15,16]
High mortality	13.2 (9.0-20.5)	Gamma (alpha = 22.99, beta = 0.62)	[15,16]
**Vaccine efficacy (ratio of one dose to last-dose)**			
Low-income and middle-income countries	0.75 (0.55-0.96)	Beta (alpha = 5.10, beta = 2.22, [A] = 0.18, [B] = 1)	Table S2
High income countries	0.94 (0.83-1.00)	Beta (alpha = 1.77, beta = 0.24, [A] = 0.50, [B] = 1)	Table S3

CI, confidence interval; RVGE, rotavirus gastroenteritis; UNWPP, United Nations World Population Prospects; U5MR, Under-five mortality rate; VE, vaccine efficacy; WUENIC, World Health Organization-UNICEF Estimates of National Immunization Coverage.

a We calculated 95% uncertainty intervals around the number of RVGE deaths prevented <5 years (2006-2019) in each country. Intervals represent the 2.5^th^ and 97.5^th^ percentiles of 100 Monte-Carlo simulations. Estimates were compared after 100, 200, 300 runs etc., and there was stability in the 2.5^th^ and 97.5^th^ percentiles after ∼100 runs.

b Other parameters of the parametric distribution were fixed during probabilistic sensitivity analysis simulations. For example, a three-parameter gamma distribution was used to estimate VE over time. Two parameters (VE after 2 weeks, mean duration) were varied in probabilistic sensitivity analysis. The third parameter (alpha) was fixed for the low (0.934), medium (0.401), and high (0.399) mortality strata.

### Rotavirus gastroenteritis mortality by country, age, and year

UNWPP 2022 revision estimates of the number of children alive in each single age and calendar year [8] were used to estimate the number of life-years lived between birth and age 5.0 years for 18 tracked birth cohorts (2002-2019) in 186 countries. For each cohort, life-years <5 years were multiplied by rates of RVGE mortality <5 years to estimate the expected number of RVGE deaths between birth and age 5.0 years. Three datasets were used to calculate an average RVGE mortality rate for each country: (i) Global Burden of Disease Study (GBD); (ii) Maternal and Child Epidemiology Estimation Group (MCEE); and (iii) the World Health Organization and Centers for Disease Control and Prevention (WHO/CDC) joint estimates. The GBD has published estimates of rotavirus deaths aged <5 years by country and year (2000-2019) as part of the GBD 2019 revision [9]. MCEE has also recently updated estimates of diarrhea deaths and rotavirus deaths in children aged <5 years by country and year (2000-2019) [10]. WHO/CDC have updated their estimates of the rotavirus fraction among diarrhea deaths aged <5 years (2000-2015) [11], and we applied these fractions to MCEE estimates of diarrhea deaths to generate updated WHO/CDC estimates of rotavirus deaths aged <5 years. For years where WHO/CDC did not report rotavirus fractions (2016-2019) we estimated them by fitting negative exponential curves to the rotavirus fractions reported in the 5-year period 2011-2015. In each country, we used the mean of the three RVGE mortality rates (GBD, MCEE, WHO/CDC) for all years before rotavirus vaccine introduction. In the absence of any basis for assessing sampling error in each of the three estimates, we calculated 95% uncertainty intervals (UIs) by treating each estimate as a distinct equally weighted sample from a population of such estimates. In countries that had introduced the vaccine before 2020, we fitted negative exponential curves to the RVGE mortality rates in the 5 years before vaccine introduction and then extrapolated this trend. This was done to estimate a counterfactual scenario of no rotavirus vaccine use in the period 2006-2019. In seven small countries, it was not possible to fit exponential curves due to small/erratic counts, so we applied a simple 5-year moving average.

Modeled estimates of RVGE deaths in children aged <5 years were assigned to each week of age using age distributions fitted to data from 92 datasets of RVGE hospital admissions in settings without rotavirus vaccination, identified in a global systematic review [12]. For countries without data, we assumed the median age distribution for the relevant under-5 mortality quintile (very low, low, medium, high, and very high). The age-specific results reported for each tracked birth cohort were later attributed to calendar years to allow annual reporting over the period 2006-2019.

### Rotavirus vaccine coverage by country, age, year, and dose

Our analysis focused on four rotavirus vaccines that were licensed for global use before 2020: Rotarix® (GlaxoSmithKline, Rixensart, Belgium), RotaTeq® (Merck, Pennsylvania, USA), Rotavac® (Bharat Biotech International, Hyderabad, India) and Rotasiil® (Serum Institute of India, Pune, India). We excluded China's domestic Lanzhou Lamb Rotavirus (LLR)® vaccine (Lanzhou Institute of Biological Products, Lanzhou, China) and Vietnam's domestic Rotavin® vaccine (PolyVac, Hanoi, Vietnam) because both vaccines were only available on the private market and there was limited information about their national coverage before 2020 [17].

WHO's online database provides information about the rotavirus vaccine product and schedule used in each country in the year 2021 [18], but not the product and schedule used in earlier years. For simplicity, we assumed the current product and schedule were used in all years of vaccination. In the small number of countries that currently use two products simultaneously, we ran a rapid search of the published and gray literature to determine the most prevalent schedule/product combination used in the period 2006-2019. The vaccine product and schedule assumptions for each country are shown in the supplementary appendix (page 7). After assuming a single product for each country, Rotarix was the most common (74 countries) followed by RotaTeq (23 countries), Rotavac (two countries), and Rotasiil (two countries).

Estimates of last dose vaccination coverage (two doses for Rotarix, three doses for RotaTeq, Rotavac, and Rotasiil) were taken from WHO-UNICEF Estimates of National Immunization Coverage (WUENIC) for the period 2006-2019 [1]. We did not account for any private market vaccine use that was not captured in these estimates. The full period from 2006 is considered to capture the impact achieved in high and middle-income countries before WHO's recommendation in 2009 to expand the use of rotavirus vaccines to all countries globally [19]. The WUENIC estimates do not report partial dose coverage for rotavirus vaccines, so we estimated early dose coverage by inflating the last dose coverage, using the drop-out ratios (dose 1:2 and dose 2:3) reported for Diphtheria, Tetanus, and Pertussis (DTP) vaccine [13]. We assumed that rotavirus vaccine doses would have the same timeliness (realistic delays in vaccine administration) as estimated for co-administered doses of DTP. Estimates of DTP vaccine timeliness were based on 71 nationally representative Demographic and Health Surveys (DHS) and Multiple Indicator Cluster Surveys (MICS) carried out between 2009 and 2016 using previously described methods [14].

We assumed that restrictions on age of vaccine administration (first dose <15 weeks, last dose <32 weeks) were removed in all scenarios to reflect the 2012 WHO Strategic Advisory Group of Experts on Immunization (SAGE) recommendation [20] but ran a separate scenario with age restrictions still in place. The difference in RVGE deaths between these two scenarios indicated the maximum potential mortality impact of the SAGE recommendation to remove age restrictions in 2012.

### Rotavirus vaccine efficacy by country, dose, and duration of follow-up

Estimates of rotavirus vaccine efficacy and waning were based on a recent Bayesian meta-analysis of 31 randomized controlled trials. We assigned different efficacy/waning assumptions to countries with low/very low, medium, and high/very high under-five mortality, using previously described methods [16]. We used the same efficacy/waning assumptions for two doses of Rotarix and two or three doses of RotaTeq, Rotavac and Rotasiil. However, because the third dose is given at older ages, the onset of waning is delayed, and this leads to marginally higher overall impact for three-dose schedules. We therefore ran a separate scenario assuming three-dose impact could be achieved in all countries using a two-dose schedule. To calculate the efficacy of one dose we multiplied the full 2/3-dose efficacy by a ratio of 0.75 (95% confidence interval 0.55-0.96) for LMICs and 0.94 (95% confidence interval 0.83-1.00) for high-income countries (HICs). These ratios were calculated by pooling data from all post-licensure studies that report rotavirus vaccine effectiveness for both the first and last dose (supplementary appendix, pages 8-9). The studies were identified from a recent global review by Burnett and colleagues [21].

### Validation analysis

We restricted our model validation exercise to LMICs, where most RVGE deaths occur. Rotavirus hospital surveillance is available in several LMICs, so we calculated the percent reduction in RVGE hospital admissions aged <5 years in each reported calendar year of rotavirus vaccination. We adjusted our estimates of vaccine impact to provide stability in the rate of test-negative gastroenteritis (GE) hospital admissions over time. For each country with an eligible surveillance dataset, we compared the surveillance-based estimates of vaccine impact to our modeled estimates. Rotavirus hospital surveillance datasets were obtained from the WHO-coordinated Global Rotavirus Surveillance Network (GRSN) [5] and the scientific literature. Full details of the search strategy, inclusion/exclusion criteria, and vaccine impact calculations are in the supplementary appendix (pages 10-15).

### Uncertainty and sensitivity analysis

In our base case scenario, we assumed the mean RVGE mortality rate from three sources (GBD, MCEE, WHO/CDC), rotavirus vaccine coverage reported by WUENIC, no age restrictions on vaccine administration, and the current product and schedule as reported by WHO for each country in all historical years. We then ran six alternative scenarios to show the effect of (a) using the GBD mortality dataset alone; (b) using the MCEE mortality dataset alone; (c) using the WHO/CDC mortality dataset alone; (d) applying age restrictions; (e) assuming all countries using the two-dose Rotarix vaccine were assigned the same impact as the three-dose vaccines (RotaTeq, Rotavac and Rotasiil); and, (f) assuming all 186 countries introduced rotavirus vaccines at DTP coverage levels over the period 2006-2019 using a three-dose vaccine.

We calculated the 2.5^th^ and 97.5^th^ percentiles of the estimated number of RVGE deaths prevented in each country and year (2006-2019) based on 100 probabilistic runs per country. Each parameter was allowed to vary within specified uncertainty ranges (Table 1).

## Results

**Figure 1:**
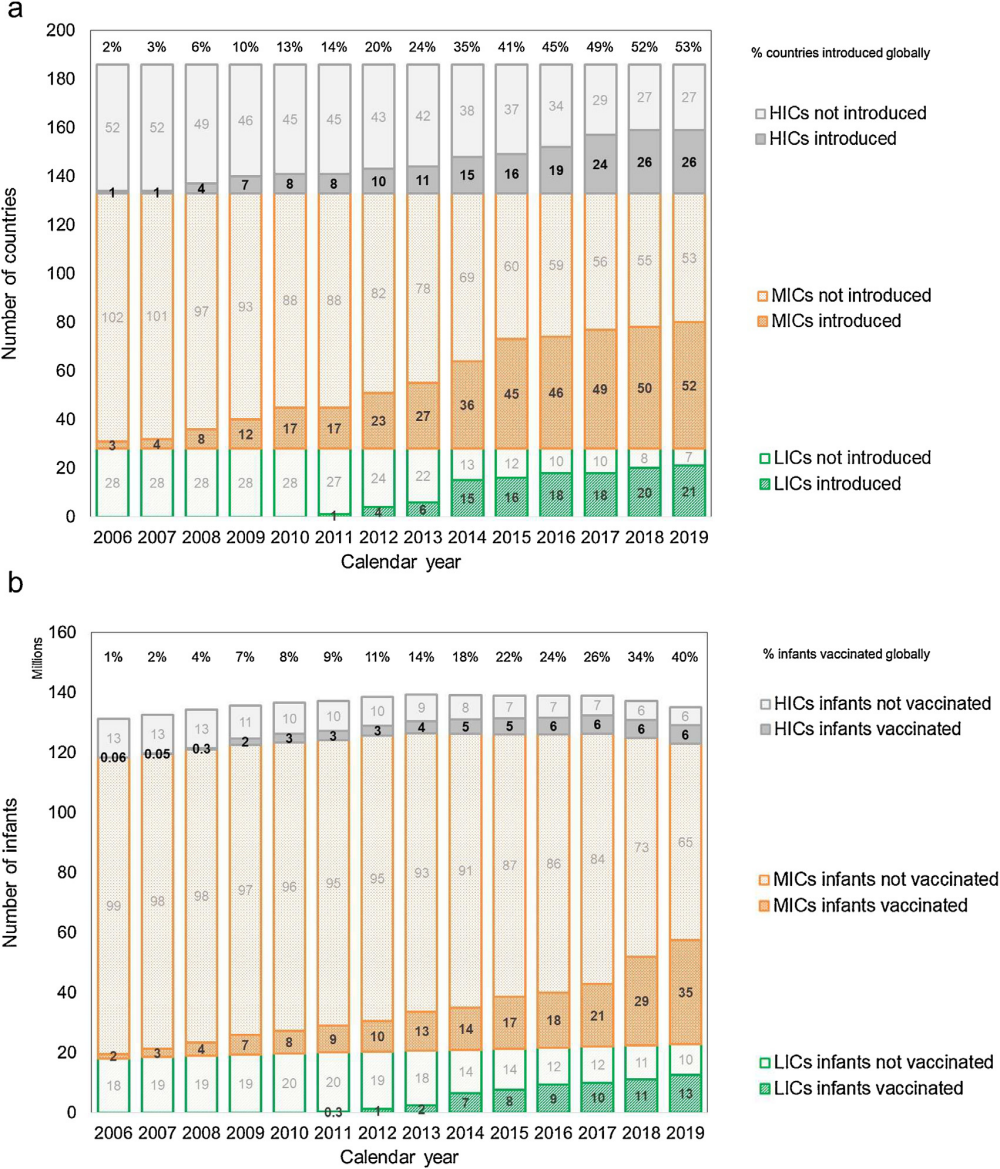
Number of countries (panel a) and infants (panel b) using rotavirus vaccines by year (2006-2019) and 2022-23 World Bank income group. HIC, high-income country; LIC, low-income country; MIC, middle-income country.

The number of countries with non-zero WUENIC rotavirus vaccination coverage increased from 2% (4/186) in the year 2006 to 53% (99/186) in the year 2019. In 2019, this represented 75% (21/28) of low-income countries (LICs), 49% (52/105) of middle-income countries (MICs), and 49% (26/53) of HICs (Figure 1**a**). The supplementary appendix (page 7) shows which countries introduced rotavirus vaccine programs, and in what sequence, over the period 2006-2019.

In the year 2019, around 54 million infants (40% of the global infant cohort) received at least two doses of rotavirus vaccination (Figure 1**b**). Around half of the 81 million infants that did not receive at least two doses were living in four MICs. India had 11 million unvaccinated infants despite 53% rotavirus vaccination coverage. In China, Indonesia, and Nigeria we assumed zero rotavirus vaccination coverage and 15, 4.5, and 7 million unvaccinated infants, respectively.

**Figure 2:**
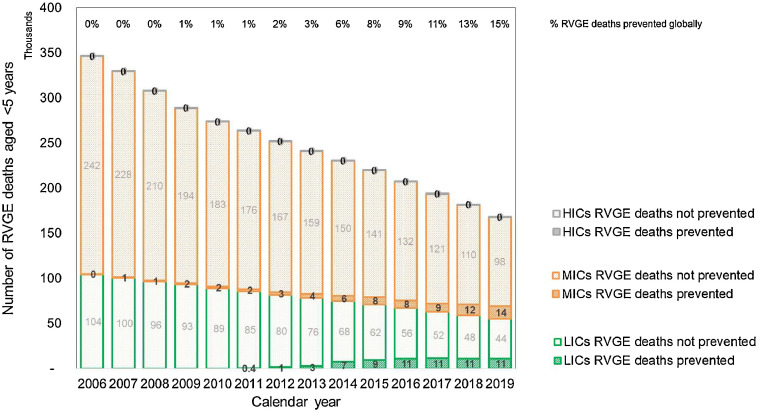
Number of rotavirus deaths prevented (and not prevented) by rotavirus vaccination in 186 countries by year (2006-2019) and 2022-23 World Bank income group. RVGE, rotavirus gastroenteritis; HIC, high-income country; LIC, low-income country; MIC, middle-income country. Caption: The overall decrease in RVGE deaths each year is due to the decreasing trend in RVGE mortality estimated by all 3 RVGE mortality datasets (Global Burden of Disease Study, Maternal and Child Epidemiology Estimation Group and World Health Organization and Centers for Disease Control and Prevention). Nine countries (Albania, Benin, Chile, Kyrgyzstan, North Macedonia, Russia, Sweden, Timor-Leste, Thailand) reported partial/restricted introduction before 2022 and two countries (China and Vietnam) both had domestic vaccines available on the private market. However, World Health Organization-UNICEF Estimates of National Immunization Coverage rotavirus vaccination coverage was 0% for the period 2006-2019 in all 11 countries, so they were excluded from the figure and analysis. The Philippines and Venezuela suspended their programs in 2016 and 2018 respectively but all other countries used rotavirus vaccine until the end of 2019. World Bank income groups for 2022-23 were applied to all historical years 2006-2019 and thus do not account for movement between income groups over time.

In the first 8 years of rotavirus vaccine use (2006-2013), global rotavirus vaccine coverage (last dose) did not exceed 15% (Figure 1**b**) and rotavirus vaccines prevented less than 5% of global RVGE deaths in children aged <5 years each year (Figure 2). In the year 2019, global rotavirus vaccine coverage (last dose) reached 40% (Figure 1**b**), and rotavirus vaccines prevented around 25,000 RVGE deaths, equivalent to 15% (95% UI 11-21%) of the global RVGE mortality burden (Figure 2) and 0.5% of the global under-five mortality burden.

Over the entire period 2006-2019, we estimate that rotavirus vaccines prevented around 139,000 deaths (95% UI 98,000-201,000) in children aged <5 years with 2,000 (1%) prevented in HICs, 72,000 (52%) prevented in MICs, and 65,000 (47%) prevented in LICs (Figure 2). In 29 countries, the total number of under-five RVGE deaths prevented by rotavirus vaccination (2006-2019) exceeded 1000 (supplementary appendix, page 16).

**Figure 3:**
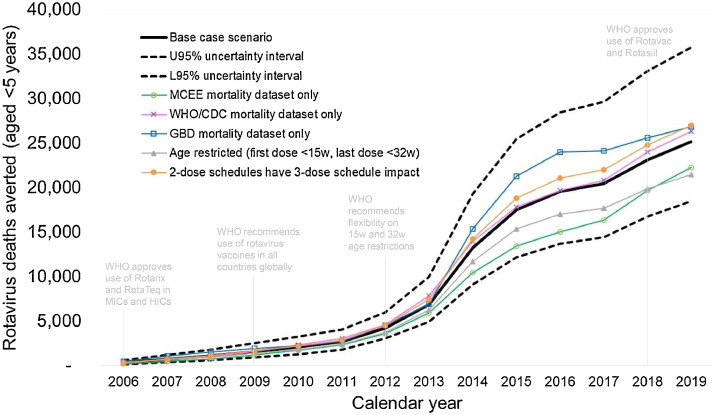
Number of RVGE deaths averted by rotavirus vaccination in 186 countries by year (2006-2019): base case (95% uncertainty interval) and alternative scenarios. GBD, Global Burden of Disease Study; MCEE, Maternal and Child Epidemiology Estimation Group; RVGE, rotavirus gastroenteritis; WHO/CDC, World Health Organization and Centers for Disease Control and Prevention. Caption: 95% uncertainty intervals are based on 100 probabilistic runs per country and include uncertainty in the population aged <5 years, RVGE mortality rates aged <5 years, RVGE age distributions, Rotavirus vaccine coverage, timeliness, efficacy, and waning. Deterministic scenarios are also shown for different mortality datasets, an age-restricted scenario and a scenario with higher impact assuming products with 2-dose schedules are assigned the same impact as products with 3-dose schedules.

We estimated around 158,000, 113,000, and 144,000 under-five RVGE deaths were prevented over the period 2006-2019 when using the GBD, MCEE, and WHO/CDC datasets, respectively. During the period 2013-2019, we estimated that 109,000 deaths would have been prevented with age restrictions vs 126,000 without them, a difference of around 17,000. If products with a two-dose schedule could achieve the same impact as those with a three-dose schedule an additional 10,000 RVGE deaths would have been prevented (149,000 vs 139,000) over the period 2006-2019 (Figure 3).

**Figure 4:**
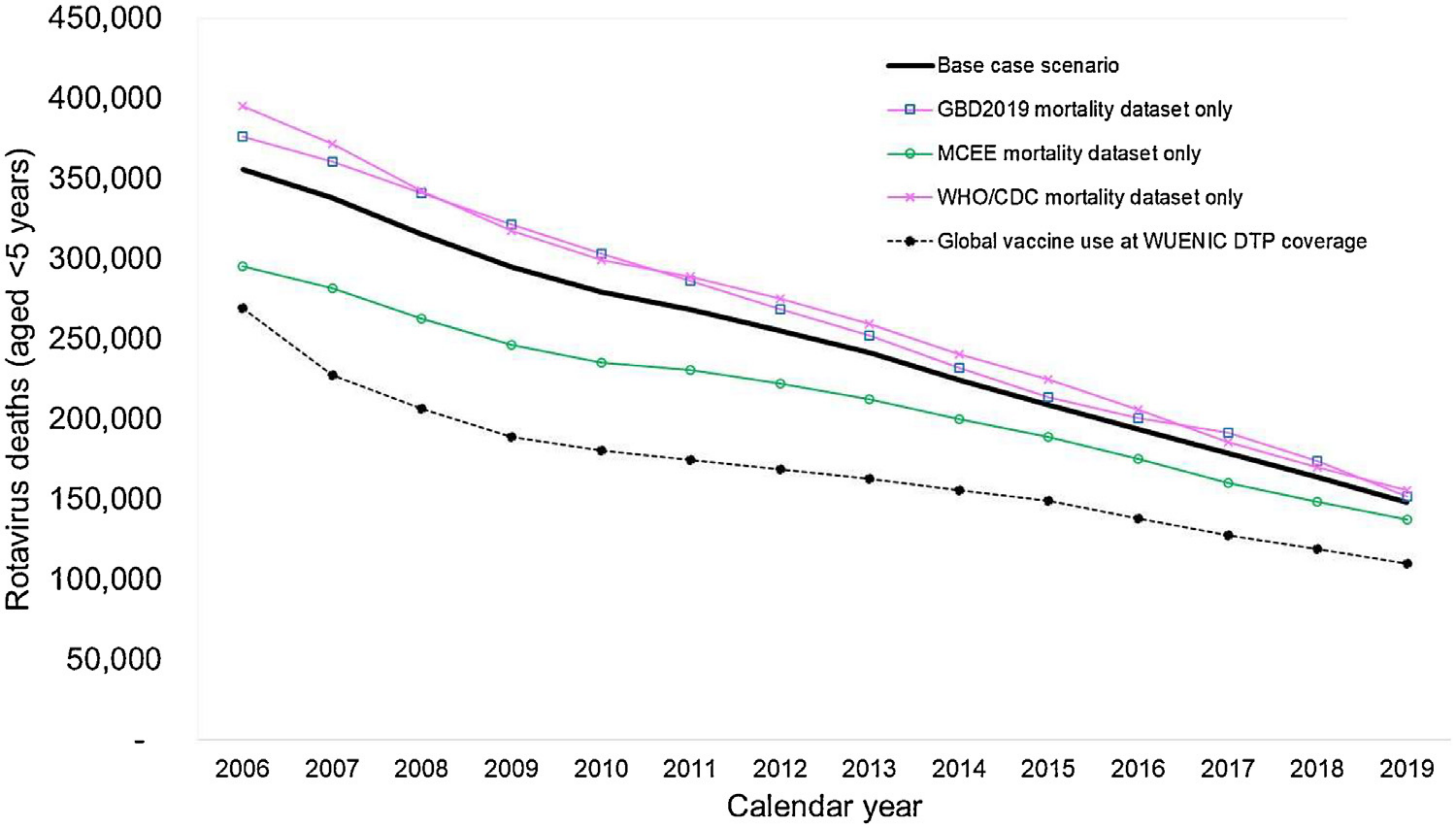
Number of rotavirus deaths in 186 countries by year (2006-2019): base case and alternative scenarios. GBD, Global Burden of Disease Study; MCEE, Maternal and Child Epidemiology Estimation Group; WHO/CDC, World Health Organization and Centers for Disease Control and Prevention; WUENIC, World Health Organization-UNICEF Estimates of National Immunization Coverage. Caption: all scenarios are adjusted for rotavirus vaccine use. The dashed black line shows the historical impact that could have been achieved had rotavirus vaccines been introduced globally in 2006 at existing Diphtheria, Tetanus, and Pertussis coverage levels. Our modeled estimates vary slightly from the official estimates produced by each mortality estimation group because we used a standard demographic dataset (United Nations World Population Prospects 2022 revision) and a standard set of 186 countries. In addition, our model tracks the experience of each birth cohort from birth to age 5.0 years then allocates estimated age-specific deaths into the appropriate calendar years post hoc.

We estimate there were around 346,000 RVGE deaths aged <5 years in 2006 but this rapidly decreased to around 143,000 in 2019. While a small proportion of this decrease was due to rotavirus vaccination, the great majority was due to a rapidly decreasing trend in all-cause diarrhea mortality over this period. Around half of the RVGE deaths estimated in the year 2019 were in three countries (DR Congo, India, and Nigeria) where rotavirus vaccine coverage (last dose) rates were 4%, 53%, and 0%, respectively. In the year 2019, we estimate that up to 63,000 RVGE deaths (37% of the global RVGE mortality burden, and 1.2% of the global under-five mortality burden) could have been prevented had all countries used rotavirus vaccines at DTP vaccine coverage levels (Figure 4).

We identified 20 LMICs with rotavirus hospital surveillance datasets (17 from the WHO GRSN and three from the literature search). Our modeled estimates were similar in eight countries, lower in nine countries, and higher in three countries (supplementary appendix, pages 17-20).

## Discussion

Our estimates highlight the important contribution that rotavirus vaccines have made to global public health. We estimate that rotavirus vaccination prevented 139,000 under-five deaths before the year 2020 with a true value likely to be in the range of 98,000 to 201,000. In 2019, around half of the RVGE deaths not prevented by vaccination were in just three countries (DR Congo, India, and Nigeria). However, between 2019 and 2021 rotavirus vaccination coverage had reportedly increased from 4 to 52% in DR Congo and from 53 to 83% in India [1], while Nigeria introduced rotavirus vaccination into its routine immunization schedule in August 2022 [22].

We estimate that rotavirus vaccines prevented less than 5% of the global rotavirus mortality burden each year in the first 8 years of their use, but this increased to around 15% in the year 2019. This was driven by the WHO recommendation to expand the use of rotavirus vaccines to all countries globally in 2009 [19] and the donor support and market shaping activities provided by Gavi, the Vaccine Alliance, which enabled many LMICs to introduce rotavirus vaccines at a low initial cost to the government.

Though rotavirus vaccines have been impactful globally, our analysis shows that the maximum potential global reduction in RVGE deaths from the direct effects of live oral rotavirus vaccines is unlikely to exceed around 40%. This is because most RVGE deaths occur in LMICs where vaccine efficacy is lower. Some improvement may be possible by optimizing the scheduling and timeliness of rotavirus vaccination programs [15], but enhanced rotavirus disease prevention and treatment strategies are needed to address the full RVGE mortality burden.

Our estimates were sensitive to the choice of RVGE mortality dataset (GBD, MCEE, WHO/CDC). One difference between these datasets is how each group takes mixed infections into account [2]. MCEE includes all diarrhea etiologies in a multinomial model that requires the fractions to add to one consistent with the principle of “one death one cause” used for cause of death estimation. GBD 2019 included attributable fractions for each cause of diarrhea death in children aged <5 years but did not assign only one etiology to each death e.g., in 2019 GBD reported 500,300 diarrhea deaths in children under 5 years of age, but the sum of the etiologic attributions totaled 653,700 [9]. The WHO/CDC joint estimates take a similar approach and do not adjust the rotavirus-attributable fraction for the presence of other enteric infections [11]. However, while this could lead to over-estimation of rotavirus deaths, studies have shown that rotavirus is usually (but not always) the dominant cause of death whenever it is detected [2]. All three groups estimated a rapid decline in RVGE deaths since 2006 largely in the absence of rotavirus vaccination likely due to increased treatment with oral rehydration therapy and zinc, and improvements in water, sanitation, nutrition, and other risk factors for diarrhea [23]. This underscores the importance of capturing realistic counterfactual (without vaccination) trends in RVGE mortality when modeling the historical or future impact of rotavirus vaccines.

Our analysis highlights the importance of the WHO SAGE recommendation to remove age restrictions in 2012 [20]. While it is not possible to say definitively what age-restriction policies were used in each of the 186 countries over the period 2006-2019, several high-mortality countries are likely to have adopted this recommendation since 2012/13. The impact of this policy will continue to grow as more LMICs introduce the vaccine and coverage improves over time.

Our analysis highlighted the importance of assumptions made about the efficacy and waning associated with two-dose and three-dose schedules. In our base case analysis, we assumed a third dose could delay the onset of waning, and therefore lead to a slightly higher impact than could be achieved with a two-dose schedule. Given that Rotarix (two doses) is the most prevalent product on the market, this was influential in our global estimates of vaccine impact. This also illustrates the importance of accurately capturing the real-world timeliness of vaccine administration and the associated delayed onset of waning. In a recent meta-analysis of the clinical benefit of the third dose [24], a small benefit was found to be moderately certain, but more evidence is needed to inform this methodological choice, particularly in LMICs [16].

Our estimates did not fully capture the broader health and economic benefits of rotavirus vaccination. While it is possible that WUENIC estimates of rotavirus vaccine coverage will have included private market use in some countries, we assume it mostly represents doses administered free of charge as part of routine national immunization programs. As a result, our analysis will have excluded the benefits associated with private market use in several countries e.g., China, Russia, and Vietnam. Finally, we did not capture the potential indirect benefits (herd effects) that may be associated with rotavirus vaccines. A recent study using a transmission dynamic model suggests that the total effect on reducing RVGE mortality (direct and indirect) could reach 49% when herd immunity is considered [25]. However, other analyses have found limited evidence of indirect effects in children aged <5 years in LMICs [26], [27], [28], and while our estimates of RVGE mortality reduction were generally conservative, we did not account for two factors that would lead to lower estimates of vaccine impact. First, we did not adjust for the potential clustering of rotavirus deaths in groups with lower vaccination coverage. Second, we assumed that reported efficacy against RVGE hospital admissions was a reasonable proxy for efficacy against RVGE deaths which may be optimistic if the data on etiologic fractions are derived from studies that excluded GE deaths associated with bloody and/or persistent diarrhea [2]. However, post-licensure data from South and Central America (e.g., Brazil, Mexico, Panama) has shown the important effect of rotavirus vaccines on overall diarrhea mortality in children <5 years old, with reductions similar to those observed for diarrhea hospital admissions [29,30]. Indeed, a recent global review of post-licensure studies published in the period 2006-2019 reported a median 36% reduction in both diarrhea mortality and diarrhea hospital admissions among children <5 years old [4].

Our model validation analysis highlighted the importance of adjusting for the expected rate of rotavirus test-negative GE hospital admissions when deriving the percent reduction in rotavirus admissions from hospital surveillance data, rather than simply estimating the percent reduction in rotavirus positivity. The latter is likely to substantially underestimate vaccine impact in some settings. Our modeled estimates were broadly consistent with surveillance-based estimates in most countries but required uncertain assumptions to be made about the rotavirus vaccination coverage in the surveillance site catchment populations.

Rotavirus vaccines have made a valuable contribution to global public health. To address the full rotavirus mortality burden, more durable and effective rotavirus vaccines (and/or other enhanced diarrhea prevention strategies) will be needed in countries with high mortality in under-5-year-old children.

## Declarations of competing interest

The authors have no competing interests to declare.

## Appendix Supplementary materials

Image, application 1

Image, application 2
