# Supplementary material for: Estimating the global impact of rotavirus vaccines on child mortality
Source: Int J Infect Dis. 2023 Dec;137:90–7. doi: 10.1016/j.ijid.2023.10.005 (PMC10689250; doi:10.1016/j.ijid.2023.10.005)
Supplement: Supplementary file 1 [file mmc1.docx]

**Supplementary appendix**

**Estimating the global impact of rotavirus vaccines on child mortality**

Andrew Clark, PhD, Sarwat Mahmud, MPH, Frederic Debellut, MSc, Clint Pecenka, PhD, Mark Jit, PhD, Jamie Perin, PhD, Jacqueline Tate, PhD, Heidi M Soeters PhD, Robert E Black, MD, Mathuram Santosham, MD, Colin Sanderson, PhD

**Countries included in the analysis**

We included 186 countries and territories defined by the 2022 revision of the United Nations World Population Prospects [1]. All countries are listed in **Table S1**. We used the World Bank 2022-23 classification to define low-income countries (LICS), middle-income countries (MICs) and high-income countries (HICs) based on Gross National Income (GNI) per capita (US$ Atlas method) thresholds of <$1,085, $1,086-13,205 and >$13,205, respectively [2].

**Table S1. Countries included the global rotavirus vaccine impact analysis**

| # | Country | World Bank income level |
| --- | --- | --- |
| 1 | Afghanistan | Low income |
| 2 | Albania | Upper middle income |
| 3 | Algeria | Lower middle income |
| 4 | Angola | Lower middle income |
| 5 | Antigua and Barbuda | High income |
| 6 | Argentina | Upper middle income |
| 7 | Armenia | Upper middle income |
| 8 | Australia | High income |
| 9 | Austria | High income |
| 10 | Azerbaijan | Upper middle income |
| 11 | Bahamas | High income |
| 12 | Bahrain | High income |
| 13 | Bangladesh | Lower middle income |
| 14 | Barbados | High income |
| 15 | Belarus | Upper middle income |
| 16 | Belgium | High income |
| 17 | Belize | Upper middle income |
| 18 | Benin | Lower middle income |
| 19 | Bhutan | Lower middle income |
| 20 | Bolivia (Plurinational State of) | Lower middle income |
| 21 | Bosnia and Herzegovina | Upper middle income |
| 22 | Botswana | Upper middle income |
| 23 | Brazil | Upper middle income |
| 24 | Brunei Darussalam | High income |
| 25 | Bulgaria | Upper middle income |
| 26 | Burkina Faso | Low income |
| 27 | Burundi | Low income |
| 28 | Cabo Verde | Lower middle income |
| 29 | Cambodia | Lower middle income |
| 30 | Cameroon | Lower middle income |
| 31 | Canada | High income |
| 32 | Central African Republic | Low income |
| 33 | Chad | Low income |
| 34 | Chile | High income |
| 35 | China | Upper middle income |
| 36 | Colombia | Upper middle income |
| 37 | Comoros | Lower middle income |
| 38 | Congo | Lower middle income |
| 39 | Costa Rica | Upper middle income |
| 40 | Côte d'Ivoire | Lower middle income |
| 41 | Croatia | High income |
| 42 | Cuba | Upper middle income |
| 43 | Cyprus | High income |
| 44 | Czechia | High income |
| 45 | Dem. People's Republic of Korea | Low income |
| 46 | Democratic Republic of the Congo | Low income |
| 47 | Denmark | High income |
| 48 | Djibouti | Lower middle income |
| 49 | Dominican Republic | Upper middle income |
| 50 | Ecuador | Upper middle income |
| 51 | Egypt | Lower middle income |
| 52 | El Salvador | Lower middle income |
| 53 | Equatorial Guinea | Upper middle income |
| 54 | Eritrea | Low income |
| 55 | Estonia | High income |
| 56 | Eswatini | Lower middle income |
| 57 | Ethiopia | Low income |
| 58 | Fiji | Upper middle income |
| 59 | Finland | High income |
| 60 | France | High income |
| 61 | Gabon | Upper middle income |
| 62 | Gambia | Low income |
| 63 | Georgia | Upper middle income |
| 64 | Germany | High income |
| 65 | Ghana | Lower middle income |
| 66 | Greece | High income |
| 67 | Grenada | Upper middle income |
| 68 | Guatemala | Upper middle income |
| 69 | Guinea | Low income |
| 70 | Guinea-Bissau | Low income |
| 71 | Guyana | Upper middle income |
| 72 | Haiti | Lower middle income |
| 73 | Honduras | Lower middle income |
| 74 | Hungary | High income |
| 75 | Iceland | High income |
| 76 | India | Lower middle income |
| 77 | Indonesia | Lower middle income |
| 78 | Iran (Islamic Republic of) | Lower middle income |
| 79 | Iraq | Upper middle income |
| 80 | Ireland | High income |
| 81 | Israel | High income |
| 82 | Italy | High income |
| 83 | Jamaica | Upper middle income |
| 84 | Japan | High income |
| 85 | Jordan | Upper middle income |
| 86 | Kazakhstan | Upper middle income |
| 87 | Kenya | Lower middle income |
| 88 | Kiribati | Lower middle income |
| 89 | Kuwait | High income |
| 90 | Kyrgyzstan | Lower middle income |
| 91 | Lao People's Democratic Republic | Lower middle income |
| 92 | Latvia | High income |
| 93 | Lebanon | Lower middle income |
| 94 | Lesotho | Lower middle income |
| 95 | Liberia | Low income |
| 96 | Libya | Upper middle income |
| 97 | Lithuania | High income |
| 98 | Luxembourg | High income |
| 99 | Madagascar | Low income |
| 100 | Malawi | Low income |
| 101 | Malaysia | Upper middle income |
| 102 | Maldives | Upper middle income |
| 103 | Mali | Low income |
| 104 | Malta | High income |
| 105 | Marshall Islands | Upper middle income |
| 106 | Mauritania | Lower middle income |
| 107 | Mauritius | Upper middle income |
| 108 | Mexico | Upper middle income |
| 109 | Micronesia (Fed. States of) | Lower middle income |
| 110 | Mongolia | Lower middle income |
| 111 | Montenegro | Upper middle income |
| 112 | Morocco | Lower middle income |
| 113 | Mozambique | Low income |
| 114 | Myanmar | Lower middle income |
| 115 | Namibia | Upper middle income |
| 116 | Nepal | Lower middle income |
| 117 | Netherlands | High income |
| 118 | New Zealand | High income |
| 119 | Nicaragua | Lower middle income |
| 120 | Niger | Low income |
| 121 | Nigeria | Lower middle income |
| 122 | North Macedonia | Upper middle income |
| 123 | Norway | High income |
| 124 | Oman | High income |
| 125 | Pakistan | Lower middle income |
| 126 | State of Palestine | Lower middle income |
| 127 | Panama | High income |
| 128 | Papua New Guinea | Lower middle income |
| 129 | Paraguay | Upper middle income |
| 130 | Peru | Upper middle income |
| 131 | Philippines | Lower middle income |
| 132 | Poland | High income |
| 133 | Portugal | High income |
| 134 | Qatar | High income |
| 135 | Republic of Korea | High income |
| 136 | Republic of Moldova | Upper middle income |
| 137 | Romania | High income |
| 138 | Russian Federation | Upper middle income |
| 139 | Rwanda | Low income |
| 140 | Saint Lucia | Upper middle income |
| 141 | Saint Vincent and the Grenadines | Upper middle income |
| 142 | Samoa | Lower middle income |
| 143 | Sao Tome and Principe | Lower middle income |
| 144 | Saudi Arabia | High income |
| 145 | Senegal | Lower middle income |
| 146 | Serbia | Upper middle income |
| 147 | Seychelles | High income |
| 148 | Sierra Leone | Low income |
| 149 | Singapore | High income |
| 150 | Slovakia | High income |
| 151 | Slovenia | High income |
| 152 | Solomon Islands | Lower middle income |
| 153 | Somalia | Low income |
| 154 | South Africa | Upper middle income |
| 155 | South Sudan | Low income |
| 156 | Spain | High income |
| 157 | Sri Lanka | Lower middle income |
| 158 | Sudan | Low income |
| 159 | Suriname | Upper middle income |
| 160 | Sweden | High income |
| 161 | Switzerland | High income |
| 162 | Syrian Arab Republic | Low income |
| 163 | Tajikistan | Lower middle income |
| 164 | Thailand | Upper middle income |
| 165 | Timor-Leste | Lower middle income |
| 166 | Togo | Low income |
| 167 | Tonga | Upper middle income |
| 168 | Trinidad and Tobago | High income |
| 169 | Tunisia | Lower middle income |
| 170 | Turkey | Upper middle income |
| 171 | Turkmenistan | Upper middle income |
| 172 | Tuvalu | Upper middle income |
| 173 | Uganda | Low income |
| 174 | Ukraine | Lower middle income |
| 175 | United Arab Emirates | High income |
| 176 | United Kingdom | High income |
| 177 | United Republic of Tanzania | Lower middle income |
| 178 | United States of America | High income |
| 179 | Uruguay | High income |
| 180 | Uzbekistan | Lower middle income |
| 181 | Vanuatu | Lower middle income |
| 182 | Venezuela (Bolivarian Republic of) | Upper middle income |
| 183 | Viet Nam | Lower middle income |
| 184 | Yemen | Low income |
| 185 | Zambia | Low income |
| 186 | Zimbabwe | Lower middle income |

**Countries excluded from the analysis**

We excluded 15 countries and territories that did not have at least one source of RVGE mortality estimates for children <5 years old (Aruba, Hong Kong, Macao, Curaçao, French Guiana, French Polynesia, Guadeloupe, Guam, Jersey, Martinique, Mayotte, New Caledonia, Reunion, United States Virgin Islands, Western Sahara), three without WUENIC [3] rotavirus vaccination coverage estimates (Kosovo, Puerto Rico, Taiwan) and several others with a population of less than 90,000 people.

**Figure S1. Rotavirus vaccine product and schedule assumptions for the period 2006-2019** *(product/schedule data from WHO[4], countries ranked by WUENIC[3] year of vaccine introduction)*

*
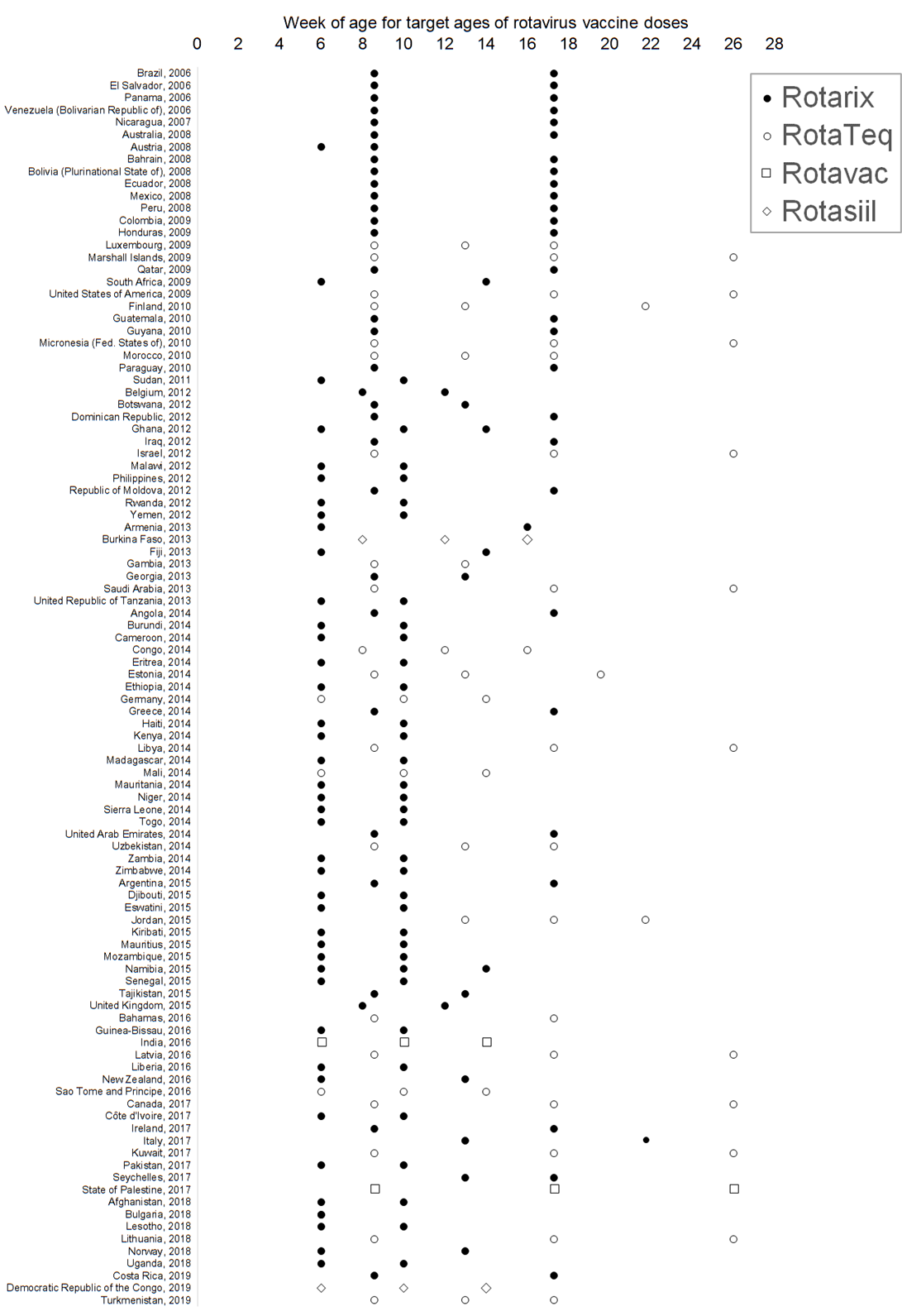
*

Caption: At least 12 countries (Australia, Belgium, Burkina Faso, Cote d’Ivoire, Germany, Greece, India, Italy, Mexico, Rwanda, State of Palestine, and USA) used multiple rotavirus vaccine products, either simultaneously or sequentially, prior to 2022. For simplicity, only one product (the most recent/common product) is shown in the figure. In the analysis of vaccine impact, this product was assumed to be in use for the entire period of vaccination.

**Table S2. Ratio of 1-dose to last-dose rotavirus vaccine effectiveness in low-income and middle-income countries**

A literature review by Burnett and colleagues [5] identified the following case control studies with estimates of 1-dose and last-dose vaccine effectiveness (VE) in LMICs. LMICs were classified using the income threshold shown above (page 2). For each pair of observations, we calculated the ratio of 1-dose to last-dose rotavirus vaccine effectiveness and generated 95% confidence intervals based on the log transformation of the effectiveness values. We calculated pooled (random effects) estimates of the 12 observations using the R package metafor [6].

| Country | Vaccine | Reference | VE (1 dose) | | | VE (last dose) | | | Ratio of 1 dose to last dose | | |  |  |  |  |
| --- | --- | --- | --- | --- | --- | --- | --- | --- | --- | --- | --- | --- | --- | --- | --- |
|  |  |  | Estimate | L95 | U95 | Estimate | L95 | U95 | Estimate | L95 | U95 |  |  |  |  |
| Bolivia1 | Rotarix | Patel et al | 36% | 0% | 59% | 69% | 54% | 79% | 0.52 | -0.32 | 1.36 |  |  |  |  |
| Bolivia2 | Rotarix | Pringle et al | 26% | 25% | 58% | 59% | 37% | 73% | 0.44 | -0.26 | 1.14 |  |  |  |  |
| Botswana | Rotarix | Gastanuday et al | 54% | -7% | 81% | 53% | 8% | 76% | 1.02 | -0.02 | 2.06 |  |  |  |  |
| Brazil | Rotarix | Ichihara et al | 60% | 37% | 75% | 72% | 44% | 85% | 0.83 | 0.41 | 1.26 |  |  |  |  |
| Burkina Faso | RotaTeq | Bonkoungou et al | 36% | -73% | 76% | 35% | -15% | 63% | 1.03 | -1.32 | 3.38 |  |  |  |  |
| El Salvador | Rotarix | de Palma et al | 51% | 26% | 67% | 76% | 64% | 84% | 0.67 | 0.25 | 1.09 |  |  |  |  |
| Kenya | Rotarix | Khagayi et al | 54% | -20% | 83% | 64% | 35% | 80% | 0.84 | -0.17 | 1.86 |  |  |  |  |
| Moldova | Rotarix | Gheorghita et al | 60% | 4% | 85% | 79% | 62% | 88% | 0.76 | 0.07 | 1.45 |  |  |  |  |
| Nicaragua | RotaTeq | Patel et al | 55% | 17% | 75% | 49% | 19% | 68% | 1.12 | 0.40 | 1.85 |  |  |  |  |
| Philippines | Rotarix | Lopez et al | 40% | -66% | 78% | 62% | 26% | 80% | 0.65 | -1.21 | 2.50 |  |  |  |  |
| South Africa | Rotarix | Groome et al | 40% | 16% | 57% | 57% | 40% | 68% | 0.70 | 0.13 | 1.27 |  |  |  |  |
| Zambia | Rotarix | Beres et al | 62% | -261% | 96% | 56% | 34% | 86% | 1.11 | -1.81 | 4.02 |  |  |  |  |
|  |  |  |  |  |  |  |  |  |  |  |  |  |  |  |  |
| **Pooled (random effects) - I^2^  = 0%** | | |  |  |  |  |  |  | **0.75** | **0.55** | **0.96** |  |  |  |  |

**Table S3. Ratio of 1-dose to last-dose rotavirus vaccine effectiveness in high-income countries**

A literature review by Burnett and colleagues [5] identified the following case control studies with estimates of 1-dose and last-dose vaccine effectiveness (VE) in HICs. HICs were classified using the income threshold shown above (page 2). For each pair of observations, we calculated the ratio of 1-dose to last-dose rotavirus vaccine effectiveness and generated 95% confidence intervals based on the log transformation of the effectiveness values. We calculated pooled (random effects) estimates of the 15 observations using the R package metafor [6].

| Country | Vaccine | Reference | VE (1 dose) | | | VE (last dose) | | | Ratio of 1 dose to last dose | | |  |  |  |  |
| --- | --- | --- | --- | --- | --- | --- | --- | --- | --- | --- | --- | --- | --- | --- | --- |
|  |  |  | Estimate | L95 | U95 | Estimate | L95 | U95 | Estimate | L95 | U95 |  |  |  |  |
| Australia1 | Rotarix | Fathima et al | 67% | 32% | 82% | 73% | 55% | 83% | 0.92 | 0.50 | 1.34 |  |  |  |  |
| Australia1 | RotaTeq | Fathima et al | 39% | -157% | 85% | 82% | 59% | 92% | 0.48 | -2.63 | 3.58 |  |  |  |  |
| Australia2* | Rotarix | Maguire et al | 60% | 53% | 67% | 70% | 66% | 72% | 0.87 | 0.74 | 1.00 |  |  |  |  |
| Canada | Rotarix | Doll et al | 93% | 69% | 98% | 91% | 61% | 91% | 1.02 | 0.80 | 1.25 |  |  |  |  |
| Israel | RotaTeq | Leshem et al | 56% | -3% | 81% | 63% | 38% | 78% | 0.89 | 0.07 | 1.70 |  |  |  |  |
| Spain | Rotarix | Martinon-Torres et al | 71% | -165% | 97% | 97% | 81% | 100% | 0.73 | -1.12 | 2.58 |  |  |  |  |
| UK | Rotarix | Walker et al | 69% | 40% | 84% | 77% | 66% | 85% | 0.90 | 0.55 | 1.24 |  |  |  |  |
| USA1 | RotaTeq | Staat et al | 89% | 16% | 99% | 95% | 48% | 99% | 0.94 | 0.40 | 1.47 |  |  |  |  |
| USA2 | Rotarix | Cortese et al | 53% | -41% | 84% | 91% | 80% | 95% | 0.58 | -0.60 | 1.76 |  |  |  |  |
| USA3 | RotaTeq | Tate et al | 51% | -38% | 83% | 84% | 71% | 91% | 0.61 | -0.59 | 1.80 |  |  |  |  |
| USA4 | RotaTeq | Donauer et al | 68% | -18% | 91% | 92% | 21% | 99% | 0.74 | -0.17 | 1.65 |  |  |  |  |
| USA5 | Rotarix | Payne et al | 57% | -45% | 87% | 70% | 39% | 86% | 0.81 | -0.39 | 2.02 |  |  |  |  |
| USA5 | RotaTeq | Payne et al | 70% | 50% | 82% | 84% | 78% | 88% | 0.83 | 0.60 | 1.07 |  |  |  |  |
| USA6 | Rotarix | Payne et al | 96% | 67% | 99% | 80% | 68% | 88% | 1.20 | 0.99 | 1.41 |  |  |  |  |
| USA6 | RotaTeq | Payne et al | 68% | 45% | 82% | 80% | 74% | 84% | 0.85 | 0.57 | 1.13 |  |  |  |  |
|  |  |  |  |  |  |  |  |  |  |  |  |  |  |  |  |
| **Pooled (random effects) - I^2^  = 18.87%** | | |  |  |  |  |  |  | **0.94** | **0.83** | **1.04^$^** |  |  |  |  |

_*_ Two periods were reported (2010-2016 and 2017). We chose the former due to the larger sample size and because 2017 was an outbreak year with a novel strain.

**^$^** The upper limit was truncated at 1.00 for use in the model.

**Model validation methods**

*Identifying rotavirus hospital surveillance datasets*

We obtained RVGE hospital surveillance datasets from two sources:

1. aggregated data from the WHO-coordinated Global Rotavirus Surveillance Network (GRSN, 18^th^ April 2023) [7]; and,
2. a literature search combining different terms for ‘rotavirus’ and ‘hospital admissions’ in PubMed, from 1^st^ January 2006 up to 10^th^ November 2022. No language restrictions were applied. The full search was as follows:

((rotavirus[MeSH Terms] OR RVGE[Text Word]) AND ((vaccin*[Text Word]) OR (immuni*[Text Word])) AND ((hospital admissions[Text Word] OR hospitalisation[Text Word] OR hospitalization[Text Word] OR admissions[Text Word] or hospital admission[Text Word])) AND (2006:2022[pdat])) AND (2006:2022[pdat]) AND (2006:2022[pdat]) AND (2006:2022[pdat])

We applied the following exclusion/inclusion criteria to all hospital surveillance datasets:

- must contain data on populations that represent LMICs;
- must contain annual data for all children aged <5 years (allowing hospital surveillance to capture any potential indirect benefits to unvaccinated children aged <5 years);
- must be possible to derive annual estimates of rotavirus positivity (proportion of GE hospital admissions that tested positive for rotavirus) as well as annual numerators (RVGE admissions) and denominators (GE admissions) in children aged <5 years;
- must allow at least two years of contiguous pre-vaccination data to be pooled (ensuring greater stability in the pre-vaccine baseline data);
- must contain at least four years of contiguous annual post-vaccination data. Four years was chosen to strike a reasonable balance between when rotavirus vaccination coverage stabilizes (typically in the second year of vaccination) and the age distribution of RVGE hospital admissions in LMICs (where >95% of RVGE admissions are aged <3 years[8]); and,
- must not have quality concerns e.g., <10 annual GE admissions tested pre-vaccination, uncertain national rotavirus vaccine coverage, implausible increase in rotavirus positivity post-vaccination.

The full search and screening process is outlined in **Figure S2** overleaf. We identified 20 LMICs with rotavirus hospital surveillance datasets (17 from the WHO-GRSN and 3 from the literature search).

**Figure S2. Search process for rotavirus hospital surveillance datasets**

*
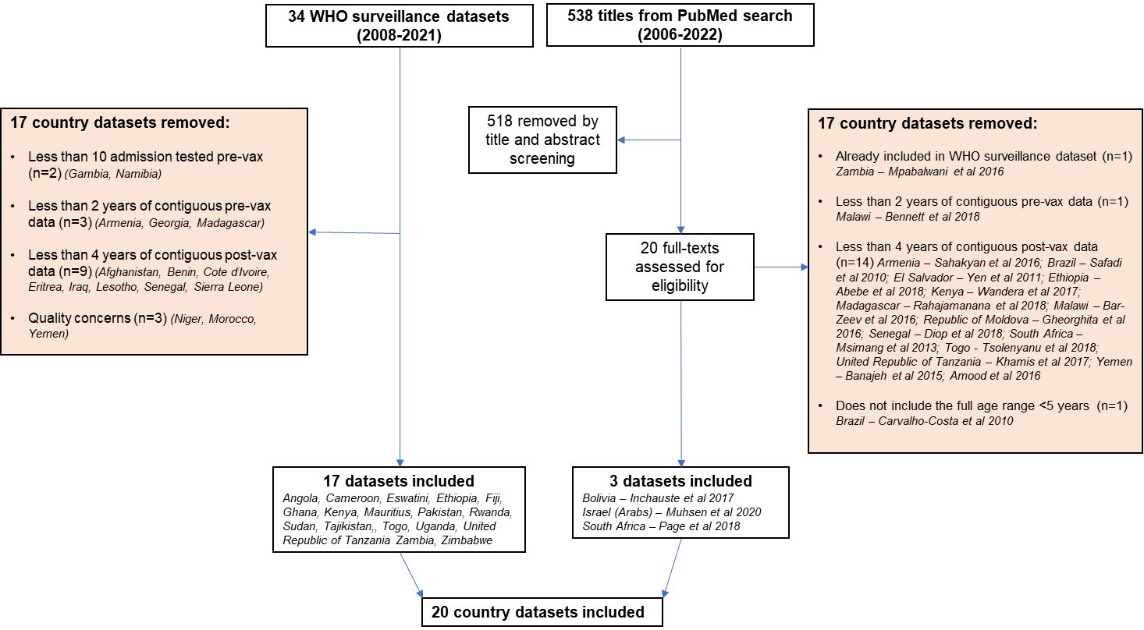
*

*Estimating the percent reduction in under-five RVGE admissions from hospital surveillance data*

Rotavirus positivity, the proportion of GE admissions that test positive for rotavirus, is commonly reported by calendar year using hospital surveillance data. As this is a reasonably stable indicator (less prone to fluctuations in total GE admissions) some authors have used the percent reduction in rotavirus positivity among children aged <5 years as a proxy for the percent reduction in RVGE admissions among children aged <5 years. This method is transparent but has the potential to under-estimate the impact of rotavirus vaccination because no adjustment is made to ensure stability in the rate of test-negative GE admissions over time.

We propose the following equation to calculate an adjusted count of RVGE admissions aged <5 years in a given post-vaccination year:

g_2_ x (( g_1_ – r_1_ ) / ( g_2_ – r_2_ )) *‘GE admissions (adjusted count in given post-vaccination year)*

x

( r_2_ / g_2_ ) *‘unadjusted rotavirus positivity in given post-vaccination year*

*where:*

r_1_ = RVGE admissions (average annual count for pre-vaccination baseline)

g_1_ = GE admissions (average annual count for pre-vaccination baseline)

r_2_ = RVGE admissions (unadjusted count in given post-vaccination year)

g_2_ = GE admissions (unadjusted count in given post-vaccination year)

As an example, there may be an average of 100 annual GE admissions pre-vaccination of which 40 are RVGE (rotavirus positivity = 40%). In a given post-vaccination year there may be 35 GE admissions of which 7 are RVGE (rotavirus positivity = 20%). To account for stability in the rate of test-negative GE admissions over time, the following equation is applied:

35 x (( 100 – 40 ) / ( 35 – 7 )) *‘75* *GE admissions (adjusted count in given post-vaccination year)*

x

( 7 / 35 ) *‘20% unadjusted rotavirus positivity in given post-vaccination year*

In the given post-vaccination year, there is now an adjusted count of 75 GE admissions. Rotavirus positivity is unchanged at 20% which gives an adjusted count of 15 RVGE admissions i.e. 75 x 20%. This approach attributes to rotavirus vaccination the prevented number of GE admissions (100 – 75 = 25) and ensures this count is equal to the prevented number of RVGE admissions (40 – 15 = 25). The % reduction in RVGE admissions in children aged <5 years is then 63% i.e. (40 – 15) / 40.

In most of the 20 LMICs with hospital surveillance datasets the rotavirus vaccine coverage stabilised after 1 year so we calculated the percent reduction in RVGE admissions in children aged <5 years in the first year, and then generated separate pooled estimates for all successive years, assuming the pooled estimate would represent each individual post-vaccination year after the first year of vaccination. This approach helped to smooth erratic fluctuations in rotavirus positivity in years when rotavirus vaccination coverage was stable. We did not include a transition year as we wanted to estimate the impact of rotavirus vaccination in all calendar years with reported rotavirus vaccination coverage.

*Comparing percent reduction in rotavirus positivity to percent reduction in RVGE admissions*

We generated plots to show both the percent reduction in rotavirus positivity and the additional percent reduction attributed to the adjustment described above. For example, in the above example the percent reduction in rotavirus positivity would be 50% i.e. (40% – 20%) / 40%. The additional percent reduction attributed to the adjustment would be 13% i.e. 63% – 50%.

*Comparing UNIVAC modelled estimates to data from hospital surveillance*

For each of the 20 LMICs with hospital surveillance datasets we compared our UNIVAC modelled estimates of the percent reduction in RVGE admissions aged <5 years in each calendar year of vaccination to the estimates derived from hospital surveillance. We populated the UNIVAC model with the best available estimates of rotavirus vaccination coverage in each calendar year of vaccination. For three countries identified in the literature we used the rotavirus vaccination coverage reported by the study in each post-vaccination year. For the 17 countries identified from the WHO GRSN, we used WUENIC rotavirus vaccination coverage at the national level for each post-vaccination year [3]. The UNIVAC model was then aligned to the reported calendar year of rotavirus vaccine introduction, and all successive years with available surveillance data. For all other UNIVAC model input parameters (e.g., demography, RVGE disease burden, RVGE age distributions, vaccine timeliness etc.) we used the country-specific inputs described in the main paper.

**Under-five RVGE deaths prevented by rotavirus vaccination (2006-2019)**

In 29 countries the number of under-five RVGE deaths prevented by rotavirus vaccination in the period 2006-2019 exceeded 1000. A ranking of these countries is shown in **Figure S3** below.

**Figure S3. Under-five RVGE deaths prevented by rotavirus vaccination by country (2006-2019)**


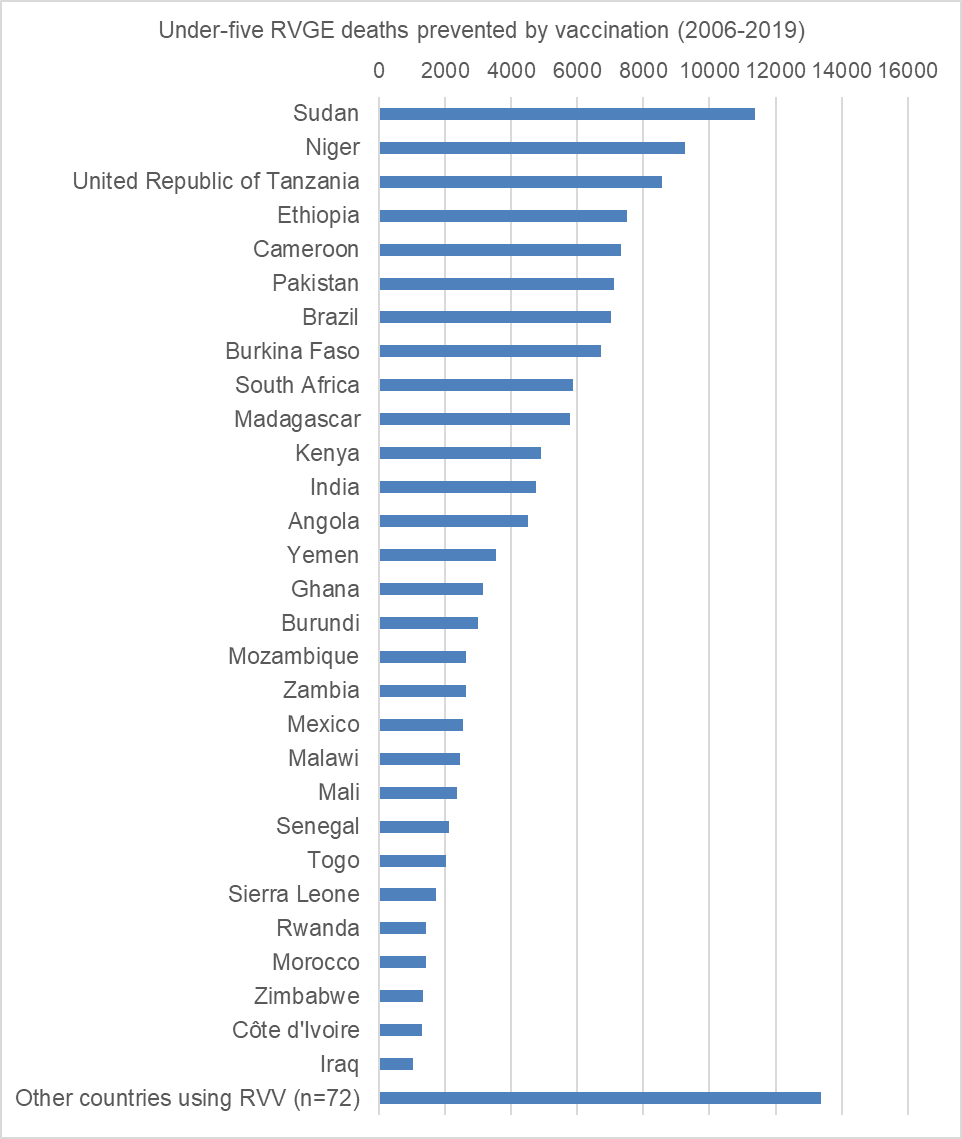


We also estimated under-five RVGE deaths prevented by rotavirus vaccination (RVV) in 72 other countries. The total number of countries with non-zero WUENIC [3] rotavirus vaccination coverage by the end of 2019 was 99/186. The Philippines and Venezuela both suspended their rotavirus vaccination programmes prior to 2019, but we estimated under-five RVGE deaths prevented in both countries during the period of rotavirus vaccine use.

**Results and limitations of model validation exercise**

*Results of model validation exercise*

Results for 17 WHO-GRSN hospital surveillance datasets are shown in **Figure S4**. Results for the three other published hospital surveillance datasets are shown in **Figure S5**. The analysis highlights the importance of adjusting for the expected rate of rotavirus test negative GE hospital admissions when deriving the percent reduction in rotavirus admissions from hospital surveillance data, rather than simply estimating the percent reduction in rotavirus positivity. This adjustment was influential in most settings. The UNIVAC modelled estimates of the percent reduction in RVGE admissions aged <5 years were similar to the surveillance-based estimates in eight countries (Ethiopia, Fiji, Mauritius, Pakistan, Tajikistan, Uganda, Bolivia and Israel-Arabs), more conservative in nine countries (Angola, Cameroon, Eswatini, Ghana, Kenya, Rwanda, Tanzania, Togo, Zimbabwe), and more optimistic in three countries (Sudan, Zambia, South Africa). It is not surprising that UNIVAC generated similar or more conservative estimates of vaccine impact than estimated by hospital surveillance in most countries (17/20) given that it is a static proportionate outcomes model capturing only the direct effects of vaccination. In the remaining three countries the reported vaccination coverage was high (>85%) but the percent reduction in rotavirus hospital admissions <5 years derived from hospital surveillance was <25% (compared to <45% in the UNIVAC model). In these countries it is possible that the rotavirus vaccination coverage in the catchment populations of the hospital surveillance sites was substantially lower than the coverage reported at the national level, but this requires further investigation. Another possible explanation is that the number of hospitals participating in the surveillance dataset changed over the period of interest in both Sudan and Zambia.

*Limitations of model validation exercise*

An important limitation of this validation analysis is the use of national WUENIC [3] rotavirus vaccination coverage (last dose) to represent rotavirus vaccination coverage in the WHO-GRSN surveillance site catchment populations. For this reason, the estimates of rotavirus vaccine impact from hospital surveillance can only be used as a crude plausibility check for the estimates generated by the UNIVAC model. For the same reason it was not possible to reliably quantify the relationship between the direct effects estimated by UNIVAC and the total (direct and indirect) effects estimated from hospital surveillance.

Another important limitation is that UNIVAC does not include country-specific estimates of vaccine efficacy and duration of protection. Instead, the model assigns efficacy/waning to each country based on a meta-analysis of several trials from the same under-five mortality stratum [9].

**
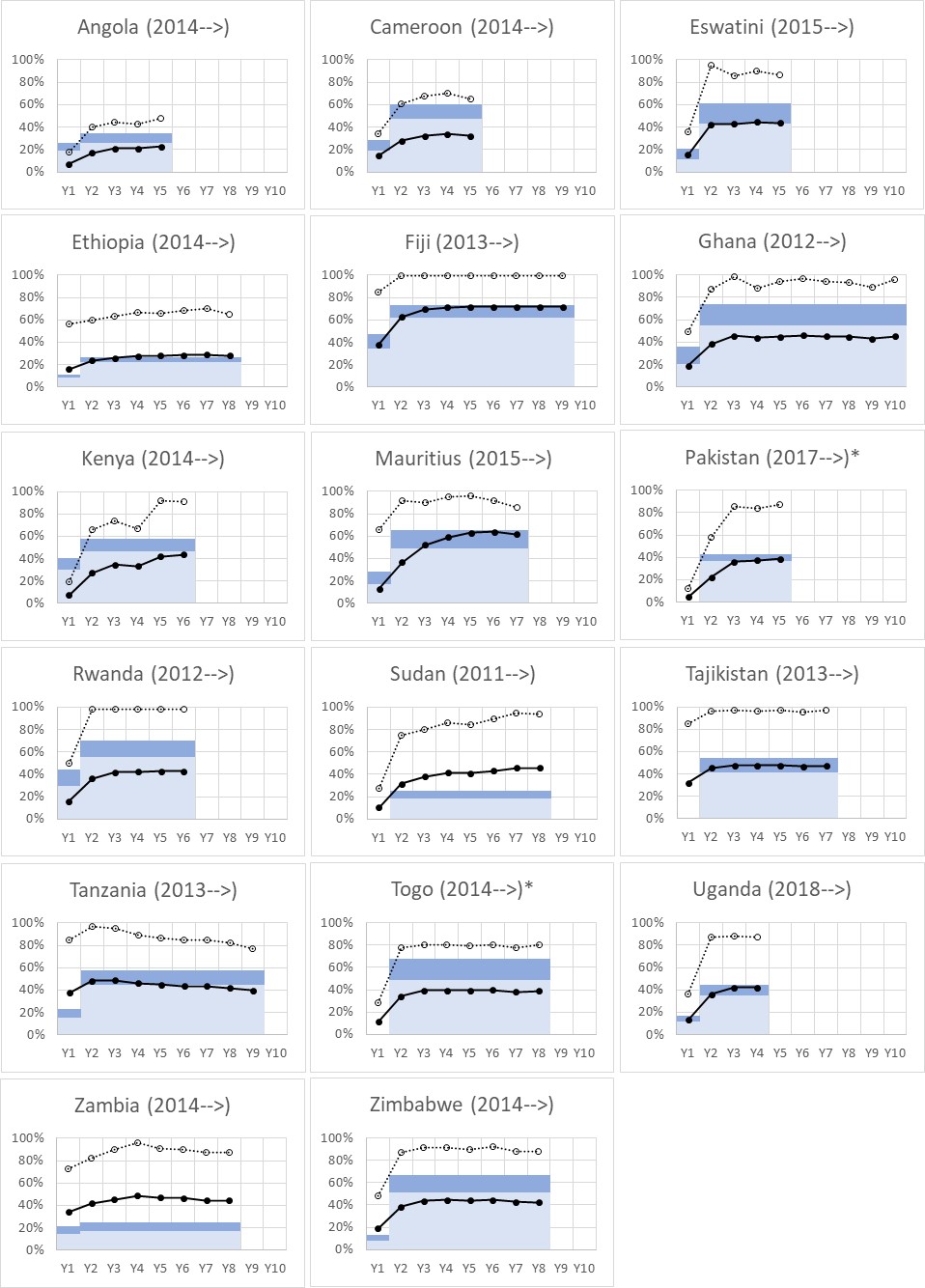
Figure S4. Estimated national WUENIC[3] rotavirus vaccination coverage (dashed black line) and percent reduction in RVGE admissions <5 years based on modelled estimates (solid black line) and sub-national WHO-GRSN[7] surveillance estimates (combined blue shaded area)**


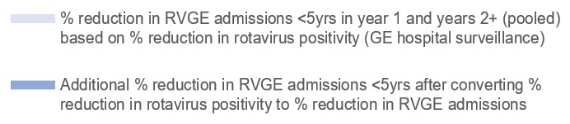

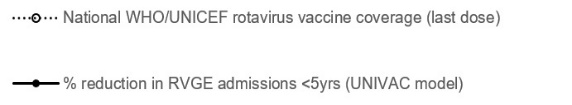


*Asterisk indicates that rotavirus vaccine introduction year has negative change in rotavirus positivity compared to pre-vaccine baseline.

**
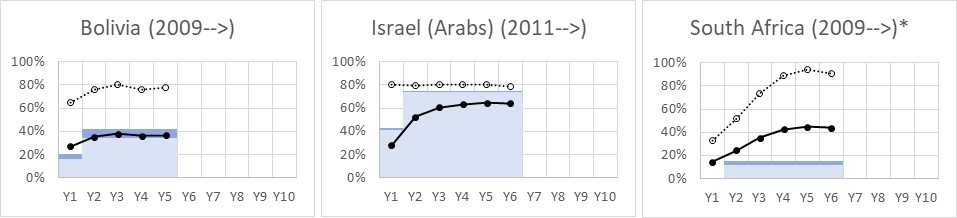
Figure S5. Estimated national WUENIC[3] rotavirus vaccination coverage (dashed black line) and percent reduction in RVGE admissions <5 years based on modelled estimates (solid black line) and other published hospital surveillance estimates (combined blue area)**


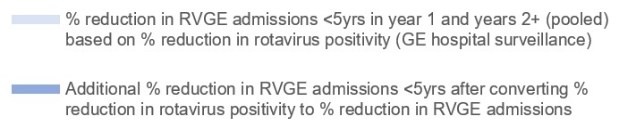

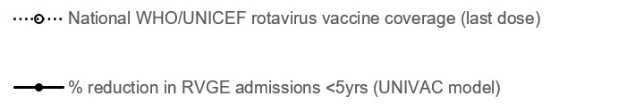


*Asterisk indicates that rotavirus vaccine introduction year has negative change in rotavirus positivity compared to pre-vaccine baseline.

**References**

[1. UNWPP. *United Nations, Department of Economic and Social Affairs, Population Division (2022). World Population Prospects 2022, Online Edition. Locations. Available at:* [*https://population.un.org/wpp/Download/Files/4_Metadata/WPP2022_F01_LOCATIONS.XLSX*](https://population.un.org/wpp/Download/Files/4_Metadata/WPP2022_F01_LOCATIONS.XLSX) *(Exported 6th December 2022).* 2022.

2. WorldBank. *New World Bank country classifications by income level: 2022-2023. GNI per capita in US$ (Atlas methodology). Available at:* [*https://blogs.worldbank.org/opendata/new-world-bank-country-classifications-income-level-2022-2023*](https://blogs.worldbank.org/opendata/new-world-bank-country-classifications-income-level-2022-2023) *(Exported 6th December 2022).* 2022.

3. WHO-UNICEF. *Rotavirus vaccination coverage. WHO/UNICEF Estimates of National Immunization Coverage (WUENIC). Updated 15th July 2022. Available at:* [*https://immunizationdata.who.int/pages/coverage/ROTA.html?CODE=Global&ANTIGEN=ROTAC&YEAR*](https://immunizationdata.who.int/pages/coverage/ROTA.html?CODE=Global&ANTIGEN=ROTAC&YEAR)*= (Exported 6th December 2022).* 2022.

4. WHO. *Vaccination schedule for Rotavirus. Available at:* [*https://immunizationdata.who.int/pages/schedule-by-disease/rotavirus.html*](https://immunizationdata.who.int/pages/schedule-by-disease/rotavirus.html) *(Exported 6th December 2022)*. 2022.

5. Burnett, E., U.D. Parashar, and J.E. Tate, *Global Impact of Rotavirus Vaccination on Diarrhea Hospitalizations and Deaths Among Children <5 Years Old: 2006-2019.* J Infect Dis, 2020. **222**(10): p. 1731-1739.

6. Viechtbauer, W., *Conducting meta-analyses in R with the metafor package* Journal of Statistical Software, , 2010. **36**(3): p. 1–48.

7. WHO. *WHO-coordinated Global Rotavirus Surveillance Network (GRSN). Excel spreadsheet containing anonymised and aggregated counts of rotavirus gastroenteritis (RVGE) and gastroenteritis (GE) hospital admissions among children aged <5 years in sentinel surveillance sites by country and calendar year.* 2023 [cited 2023 18th April]; Available from: <https://www.who.int/teams/immunization-vaccines-and-biologicals/immunization-analysis-and-insights/surveillance/surveillance-for-vpds/>.

8. Hasso-Agopsowicz, M., et al., *Global Review of the Age Distribution of Rotavirus Disease in Children Aged <5 Years Before the Introduction of Rotavirus Vaccination.* Clin Infect Dis, 2019. **69**(6): p. 1071-1078.

9. Clark, A., et al., *Efficacy of live oral rotavirus vaccines by duration of follow-up: a meta-regression of randomised controlled trials.* Lancet Infect Dis, 2019. **19**(7): p. 717-727.
